# F142. THE USE OF NEUROIMAGING MARKERS IN STRATIFIED DIAGNOSIS AND THERAPY OF SCHIZOPHRENIC AND AFFECTIVE DISORDERS

**DOI:** 10.1093/schbul/sby017.673

**Published:** 2018-04-01

**Authors:** Oliver Gruber

**Affiliations:** University Clinic Heidelberg

## Abstract

**Background:**

Neuroimaging techniques have been developed as important tools to investigate brain dysfunctions that underlie mental disorders. In particular, modern functional magnetic resonance imaging (fMRI) holds the promise to provide neurofunctional biomarkers for improved diagnosis, prognosis, and optimized treatment of schizophrenic and affective disorders.

**Methods:**

Neurofunctional connectivity MRI using advanced experimental paradigms permits targeted investigation of the functional integrity of brain systems involved in the pathomechanisms of schizophrenic and affective disorders. From these investigations, pathophysiologically relevant neuroimaging biomarkers can be derived for differential diagnosis and tailored treatment selection.

**Results:**

Possible neuroimaging biomarkers will be presented for the prediction of development and clinical course of schizophrenic and affective disorders as well as for the prediction of individual treatment responses. Further, recent neuroimaging findings on possible pathophysiological subtypes of schizophrenic and affective disorders will be discussed.

**Discussion:**

These findings from functional neuroimaging studies may help to foster the development of precision medicine in psychiatry.

